# Efficacy of zinc and copper oxide nanoparticles as heat and corrosion-resistant pigments in paint formulations

**DOI:** 10.1038/s41598-024-74345-0

**Published:** 2024-10-18

**Authors:** H. Abd El-Wahab, Ebtsam K Alenezy, Noha Omer, Mahmoud A. Abdelaziz, Rasha Jame, Shareefa Ahmed Alshareef, M. E. Owda

**Affiliations:** 1 Chemistry Department, Faculty of Science (Boys), Al-Azhar University, Cairo, Egypt; 2 Chemistry Department, College of Science, Jouf University, Sakaka, Aljouf, 72341, Saudi Arabia; 3 Department of Chemistry, Faculty of Science, University of Tabuk, , Tabuk, 71491 Saudi Arabia; 4 Department of Chemistry, Alwajh College, University of Tabuk, Tabuk, 71491 Saudi Arabia

**Keywords:** Protective paints, Heat resistant paint, Corrosion resistance paint, Pigments, Zinc oxide and copper oxide NPs, Electrochemistry, Green chemistry, Materials chemistry

## Abstract

This study focuses on the synthesis of zinc and copper oxide nanoparticles using green methods by plant extracts. The resulting metal oxides were analyzed using FT-IR spectroscopy, TGA, TEM, zeta potential and assessed for their efficacy as pigments based on properties such as Hydrogen Ion Concentration, Oil absorption, Moisture Content, Fineness of grinding, Bleeding, and loss on ignition. The results confirmed that the prepared ZnO and CuO nanoparticles exhibited the formation of nanoparticles in the range of 10–40 nm with potential as pigments. Two paint formulations incorporating these nanoparticles and silicon resins as binders were tested for physico-mechanical attributes, chemical resistance, heat resistance, and corrosion resistance of the dry paint films. The study found that the films containing the prepared oxides demonstrated excellent performance, with no damage or color alteration observed after exposure to temperatures up to 500 °C. Moreover, the paint films containing ZnO nanoparticles showed superior efficiency after a 500 h salt spray test compared to those with CuO nanoparticles. These findings suggest that the synthesized mixed oxide nanoparticles are promising candidates for heat-resistant pigment applications.

## Introduction

Ensuring the protection of outdoor carbon steel equipment, including items like exhausters, furnaces, BBQ chimneys, grills, and ovens, poses a considerable challenge, particularly when it comes to applying decorative coatings. These coatings must not only enhance aesthetics but also perform well under varying weather conditions. Specifically, they need to exhibit both heat resistance and corrosion resistance. Moreover, coatings used on cooking gadgets must also comply with indirect food safety laws. High-temperature coatings are specifically formulated to preserve their barrier properties even when exposed to aggressive environmental conditions at temperatures exceeding 1200 °C. Among these coatings, Silicon-based coating materials are of particular interest in the realm of protective coatings for high-temperature applications because of their favorable thermal stability features^1–3^. Polysiloxane is the binder often employed in coatings for high-temperature applications. Its unique properties, such as excellent thermal stability and resistance to extreme temperatures, make it well suited for protective applications in challenging environments^4^. Researchers have often modified the polymer backbone by incorporating aromatic and aliphatic groups to enhance thermal stability, and it has been studied for its applications at elevated temperatures^5,6^. Zinc-rich coatings, containing zinc powder, provide excellent cathodic protection to steel substrate. After the coating is cured, zinc imparts electrical conductivity to the matrix. Zinc particles are attacked preferentially by the corrosive medium during the early stages of coating performance, which ensures cathodic protection for the steel substrate^7,8^. Zinc-rich inorganic coatings, which utilize an ethyl silicate binder, fall under the category of high-performance coatings. They are specifically designed to safeguard steel from corrosion in challenging environments, including underground, marine, and industrial settings, as well as in nuclear power plants. These coatings provide great resistance against corrosion even at temperatures high as 400 °C for steel substrates^9,10^. Although there are numerous heat-resistant coatings and coatings designed for corrosion protection, only a few studies have specifically addressed the combination of both criteria^11–14^.

The study also examined the prediction of heat accumulation in solar reflecting coatings, based on the physico-chemical properties of complex inorganic color pigments^15^. Heat-resistant coatings play a crucial role in industrial applications, particularly in equipment such as reactors, exhaust pipes, spacecraft, and stacks that experience permanent or occasional exposure to elevated temperatures. When formulating heat-resistant coatings, adhering to specific restrictions is a challenge. Notably, pigments that sublime, decompose, or change color under heat are clearly inappropriate. Consequently, this limitation effectively rules out most organic and inorganic pigments, although some may withstand moderate heat; they fail completely at higher temperatures^16^. Anticorrosive spinel structures have been extensively studied over the years. Typically, they are heat stable, high-quality, heat-resistant pigments come in various shades and are well-suited for demanding applications. Notably, anticorrosive pigments fall within this crucial category of inorganic pigments and are known for their inhibiting effect when applied to paints^17^. A selection of nanosized mixed metal oxides were synthesized and assessed as pigments with exceptional heat resistance^18,19^. These pigments exhibited good corrosion resistance (in 5% NaCl for 500 h) and heat resistance (up to 600 °C). The synthesis techniques used for these pigments are simple and cost-effective, making them suitable for various industrial applications^20^. Additionally, recent studies have explored nontoxic anticorrosive pigments based on iron oxide and divalent metal oxides in ferrites, which offer improved corrosion protection^21^. However, further research is required to develop more efficient nanosized pigments for the coating industry. Over the last decade, researchers have well known a fascinating category of coatings known as organic-inorganic hybrids. These coatings combine the advantages of organic and inorganic materials to produce nanocomposites with unique properties. By incorporating low-cost inorganic particles into resins, these hybrids offer improved anticorrosive, thermal, and mechanical properties, as well as enhanced wear resistance. Notably, these inorganic materials are renowned for their heat and corrosion resistance^22^. Coatings made from silicon resin can withstand temperatures up to 600 °C, but their high cost poses a challenge. To address this, researchers propose developing novel heat-resistant coatings that maintain high-temperature performance while being more cost-effective^23,24^. The study reported the utilization of nano-magnesium oxide (MgO NPs) and nano-zinc oxide (ZnO NPs) as flame retardants and anticorrosion agents. Various tests including AFM, XRD, FT-IR, combustion test, thermal conductivity, thermal gravimetric decomposition, corrosion rate, hardness, and tensile strength were conducted to evaluate their effectiveness^25^. A new approach was used to introduce high-temperature binders, namely Al(H_2_PO_4_)_3_ solution and silica sol. At high temperatures, they transformed into macromolecular polymer chains, network architectures, and SiO_2_ particles, resulting in both high-temperature stability and adhesion. A study was conducted to investigate the impact of several types of silica sol, additives, and functional fillers on the corrosion resistance of the coating. The ecologically friendly inorganic insulating coating, when cured at 475 ^o^C and annealed at 800 ^o^C, exhibits exceptional corrosion resistance. This coating is compatible with the existing rolling process of orientated silicon steel. The salt-spray resistance can endure for a minimum of 24 h and a maximum of 72 h^14^. New heat- and corrosion-resistant coating methods, suitable for use in outdoor areas where there is indirect contact with food, have been created. Two systems were developed to address the limitations of traditional heat-resistant surface-protective solutions for outdoor cooking equipment. The first system is a single-layer, polysiloxane-based solution that is oven-dried. The second system is a two-layer solution that contains zinc phosphate active pigment and is cured at ambient temperature^26^. The study aims to examine and compare the anticorrosive characteristics of PANI/Zn and PANI/epoxy/Zn nanocomposite coatings to assess the impact of the epoxy component on the corrosion resistance of the PANI/Zn coating^27^. A study was conducted to prepare modified alkyd and PEA nanocomposite binders using bio ZnO and bio CuO/ZnO nanoparticles. The materials were then analyzed for their corrosion resistance, physico-mechanical characteristics, and chemical resistance properties. The results showed that the newly developed materials exhibited unique physical and mechanical properties, as well as promising corrosion-resistance properties when applied to steel substrates exposed to a saline corrosive medium. Additionally, double-cation MMOs pigments (CaMnO_3_ and Ca_2_Cr_2_O_6_ compounds) were successfully prepared using solid-state calcination and co-precipitation methods. These oxides exhibit good heat resistance (up to 600 °C) and corrosion resistance. In silicone resin-based paints, the optimal pigment mass loading ratio was found to be 2:1 (resin to pigment), a recommended by the silicone resin manufacturer and confirmed through resin property analysis after oxide pigment additions^20^. Multifunctional coatings are designed to respond effectively to external environmental conditions and provide multiple functions within a single coating. In this study, a cost-effective coating system was developed by blending an expensive resin (silicon) with a more affordable resin (alkyd) in a 4:1 ratio. The presence of the cation-exchanged P-zeolite enhanced the heat and corrosion resistance of the system. Various zeolite materials, including Na–P-Ze, Cu–P-Ze, Zn–P-Ze, and (Zn.Cu)–P-Ze, were investigated for their synergistic effects in practical applications^12^.

Thermal coatings are advanced materials applied to metallic surfaces in high-temperature environments, such as gas turbine parts and automotive exhaust systems. These coatings insulate components from prolonged heat loads, allowing for higher operating temperatures while reducing oxidation and thermal fatigue. However, due to differences in thermal expansion between the substrate and coating, cracks may form, leading to a hierarchical structure^28^. Organic coatings play a crucial role in protecting metal substrates from corrosion. They achieve this through two primary mechanisms:

1. *Electrochemical Passivation* Anticorrosive pigments within the coating inhibit corrosive reactions. By sharing these pigments, the coating forms a stable and strongly adhesive layer on the metal surface. This passivation process helps prevent aggressive agents (such as water and oxygen) from reaching the metal.
2. *Barrier Mechanism* The coating acts as a barrier, limiting the access of corrosive environmental elements to the metal surface. This protective function helps extend the lifespan of metal components.

Additionally, spinel pigments - characterized by the general formula AB_2_O_4_ - contribute to these coatings. Their almost cubic oxygen arrangement accommodates cations at tetrahedral and octahedral sites. These spinel pigments exhibit high hardness and color stability^29^.

The aim of this study is to examine and compare the effects of metal oxide nanoparticles as Heat and Corrosion-Resistant Pigments in Paint Formulations.

## Experimental procedure

All the employed resins, extenders, additives, solvents, and chemicals were fine chemical grades and were products of different international and local companies. Chemically pure grade calcium Carbonate (CaCO_3_) was purchased from Merck Germany. Iron Trioxide (Fe_2_O_3_) is a product of Sensient chemicals Co. England. Zinc acetate and copper nitrate were purchased from a pioneer (Chemicals-Piochem, Egypt).

### Preparation of NPs

**Fig. 1:**
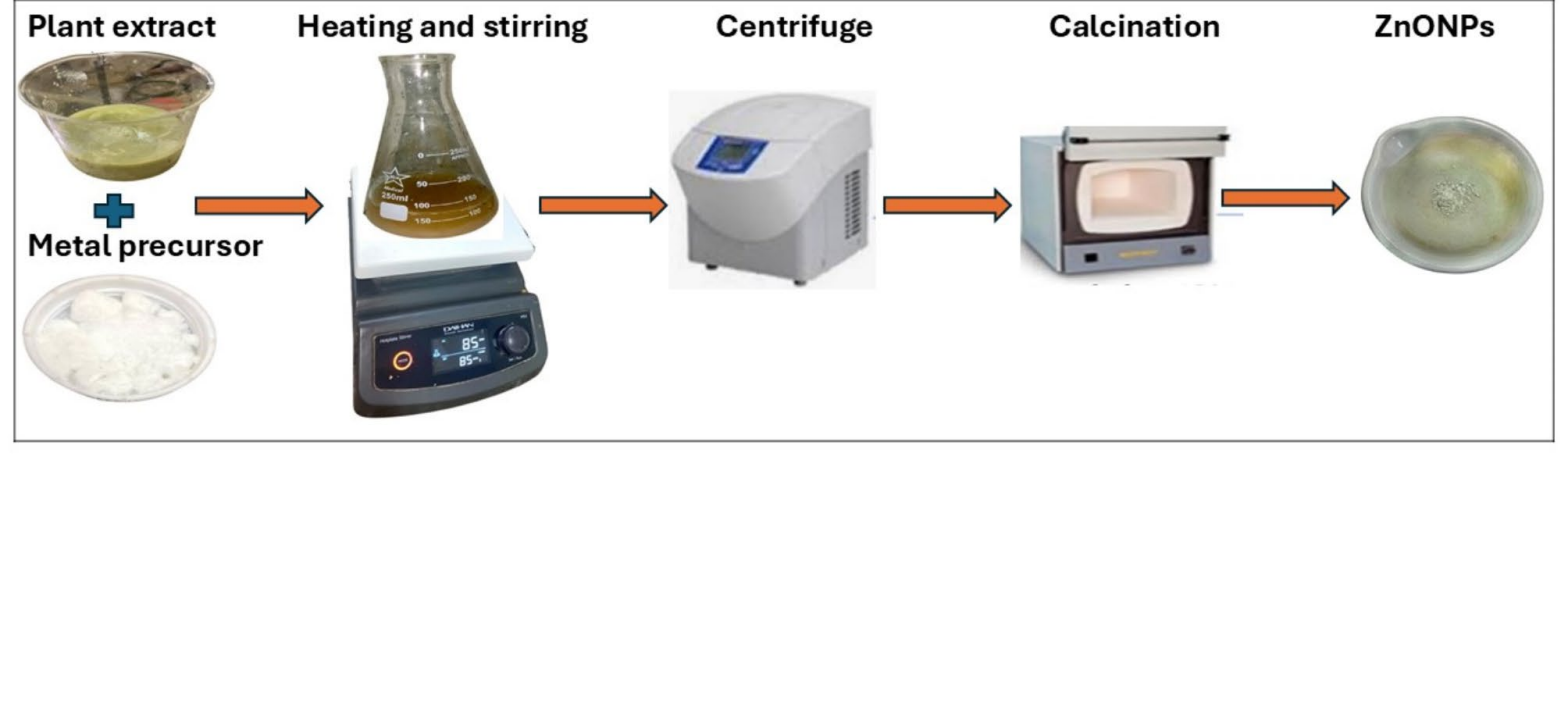
Schematic diagram for the green synthesized nanoparticles.

The formation of nanoparticles using plant extract is illustrated in Fig. 1. As shown, okra extract (100 mL) was added separately to 100 ml of 1mM zinc acetate and 100 ml of 1mM copper nitrate solution while stirring at room temperature. The temperature was then raised to 55 °C until a light-yellow and brown color appeared for ZnO and CuO NPs respectively. The nanoparticles were separated by centrifugation at 10,000 rpm for 5 min and subsequently calcined at 450 °C for 3 h to obtain pure metal oxide nanoparticles.

### Method of instrumental analysis

#### FT- IR spectroscopy

Fourier-transform infrared (FTIR) spectra were obtained using a Bruker (Vector 22) single-beam spectrometer with a resolution of 4 cm^−1^. The specimens were pulverized using potassium bromide (KBr) in a ratio of 1:100, forming a tablet. These tablets were then affixed to the sample holder located within the spectrometer’s cavity. The measurements were taken at ambient temperature within the range of 400 to 4000 cm^−1^.

#### Thermogravimetric analysis

Thermogravimetric analysis, a Perkin Elmer thermal analysis (DSC 6000, USA) the temperature increased at a rate of 5 °C per minute under a nitrogen atmosphere.

#### Transmission electron microscope (TEM)

The samples were examined using transmission electron microscopy (TEM) on a JEOL (GEM-1010) instrument at a voltage of 76 kV.

#### Scanning electron microscope (SEM)

A JEOL JSM-T 330A scanning electron microscope operating at an acceleration voltage of 30 kV was used to investigate the physical properties of the precipitated powder.

#### Hydrodynamic size distribution and zeta potential

Particle size and zeta potential of the prepared NPs were measured using a Zeta-Sizer (Malvern, UK).

#### Pigment evaluation

The prepared pigments were evaluated for their oil absorption (ASTM D281–95, 2007), bleed test (ASTM D 279–87, 1997), moisture content (D 280–95), hydrogen ion concentration (pH value) (ASTM D 1583–01, Fineness of Dispersion 1210–96, and loss of ignition (ASTM D1208).

## Techniques

### Paint preparation

Two paint formulations, containing ZnO and CuO NPs as pigments and silicon resins as binders, were prepared. The paints were formulated with a pigment-to-binder ratio of 2:1 and were prepared using a ball mill. Subsequently, the corrosion and heat resistance of the coated films were compared. The formulations based on the ZnO and CuO nanoparticles are listed in Table 1. The steel surface was prepared following ASTM D609-00 (Procedure D: solvent wiping), and the paints were applied using a film applicator with a thickness of 80 ± 5 μm^12^.

**Table 1:** Illustrates the heat-resistance paint formula including the prepared pigments.

Ingredients	F1	F2	F3	F4
Silicone resin	55	55	55	55
Silica Fumed	3	3	3	3
Zinc phosphate		25		
The prepared ZnO NPs			25	25
The prepared CuO NPs				
Talc	25			
Butyl glycol	4	4	4	4
Xylene	13	13	13	13
Total	100%	100%	100%	100%

Total pigment: 56.

Total P / B: 2: 1.

### Methods of testing and evaluation of the coated films

#### Physical and mechanical tests

Several physical and mechanical assessments of the paint films were conducted. The sample preparation and evaluations encompassed pertinent methodologies. The process of preparing the steel panels D609-17. Quantification of the thickness of a film in accordance with the ASTM standard method D1005-13. The specular gloss measurements were conducted in accordance with the ASTM technique D523-18, while the film hardness was assessed using a pencil hardness tester following the ASTM method D3363-11. Adhesion was assessed using a cross-hatch cutter, following the guidelines of ASTM method D3359-17. Flexibility was measured in accordance with ASTM method D522-17. The standard test method D 2794–93 (Reapproved 2001) measures the resistance of organic coatings to the effects of rapid deformation, specifically impact.

#### Heat resistance test

The ASTM D248the 5–91 shows test methods for evaluating the heat-resistant coating designed to protect steel surfaces exposed to high temperatures environments. Two methods were described: Method A for interior service coatings and Method B for exterior service coatings. The panels were positioned in a muffle furnace and maintained at various times and temperatures, as indicated in Table 2.

**Table 2:** Results of the heat resistance test at different times and temperatures.

Time	6 h	6 h	6 h	6 h	6 h	3 h
Temperature	225 °C	250 °C	300 °C	350 °C	400 °C	500 °C

#### Corrosion tests

Summary of corrosion tests conducted on the coated steel panels.

1. Salt Spray Exposure (ASTM B117-03): The coated panels were placed in a salt spray cabinet at 35 °C with 100% humidity. The test solution consisted of 5% NaCl in water, with an exposure duration of 500 h.
2. Degree of Rusting (ASTM D6294-98, 2007): Researchers evaluated the amount of visible surface rust using a standardized zero-to-ten scale. Rust distribution was classified as spot rust, general rust, pinpoint rust, or hybrid rust.
3. Degree of Blistering (ASTM D714-07, 2007): The size and density of blisters formed on the painted steel surfaces were assessed. This helps compare the severity of blistering due to corrosion.
4. Filiform Corrosion Resistance Inspection: Photographic inspection and grading of paint films were performed using standard methods. This evaluation focused on the filiform corrosion resistance.
5. Scribe Failure (ASTM D1654-92, reapproved 2000): The adhesion loss at a scribe mark or other film failure was determined for painted or coated specimens subjected to corrosive environments.

## Results and discussion

### Characterization of the prepared ZnO and CuO NPs

#### Infrared spectra of NPs

**Fig. 2:**
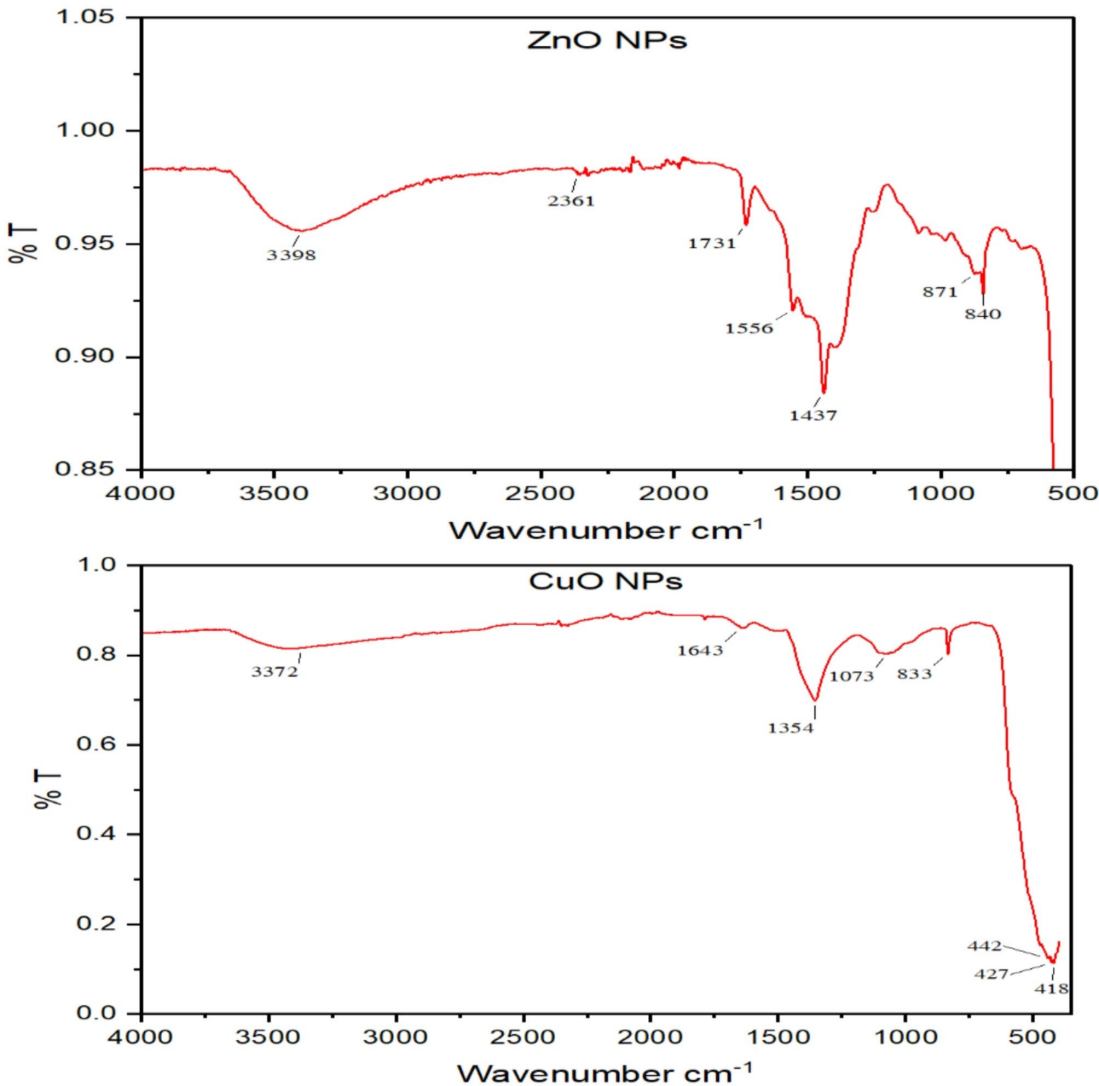
FT-IR analysis of the green-synthesized ZnO and CuO NPs.

FT-IR spectroscopy was used to identify the main bonds in the green-synthesized ZnO and CuO NPs. In Fig. 2, the FT- IR spectrum of ZnO nanoparticles displays a peak at 871 cm^−1^ which is associated with Zn–O stretch. The absorption peak at 3398 cm^−1^ is associated with the stretching vibration of the –OH group while the peak at 2361 cm^−1^ maybe be attributed to atmospheric CO_2_^30,31^. The presence of the ester’s C=O stretch bond is confirmed by the absorbance peak at 1732 cm^−1^ confirms the presence of the C=O stretch bond of ester, and the peak at 1556 cm^−1^ is related to the C=C stretch bonds of cyclic alkene^32^. The spectrum of CuO NPs reveals peaks at 3372, 1354 cm^−1^ corresponding to –OH stretching of water surrounding in the copper oxide. The Peak at 1073 cm^−1^ indicate the presence of C–O stretching frequency, while the peak at 1643 cm^−1^ indicates an unreacted ketone group, suggests the presence of flavanones adsorbed on the CuO nanostructures’ surface^33^, a long with peaks at 1073, 833, 472, 442, 427, and 418 cm^−1^. The characteristic peaks of CuO range from 418 to 472 cm^−134,35^.The band at 820 cm^−1^ may be due to stretching vibration of the Cu–O–Cu bond^36^.

#### Thermogravimetric analysis

**Fig. 3:**
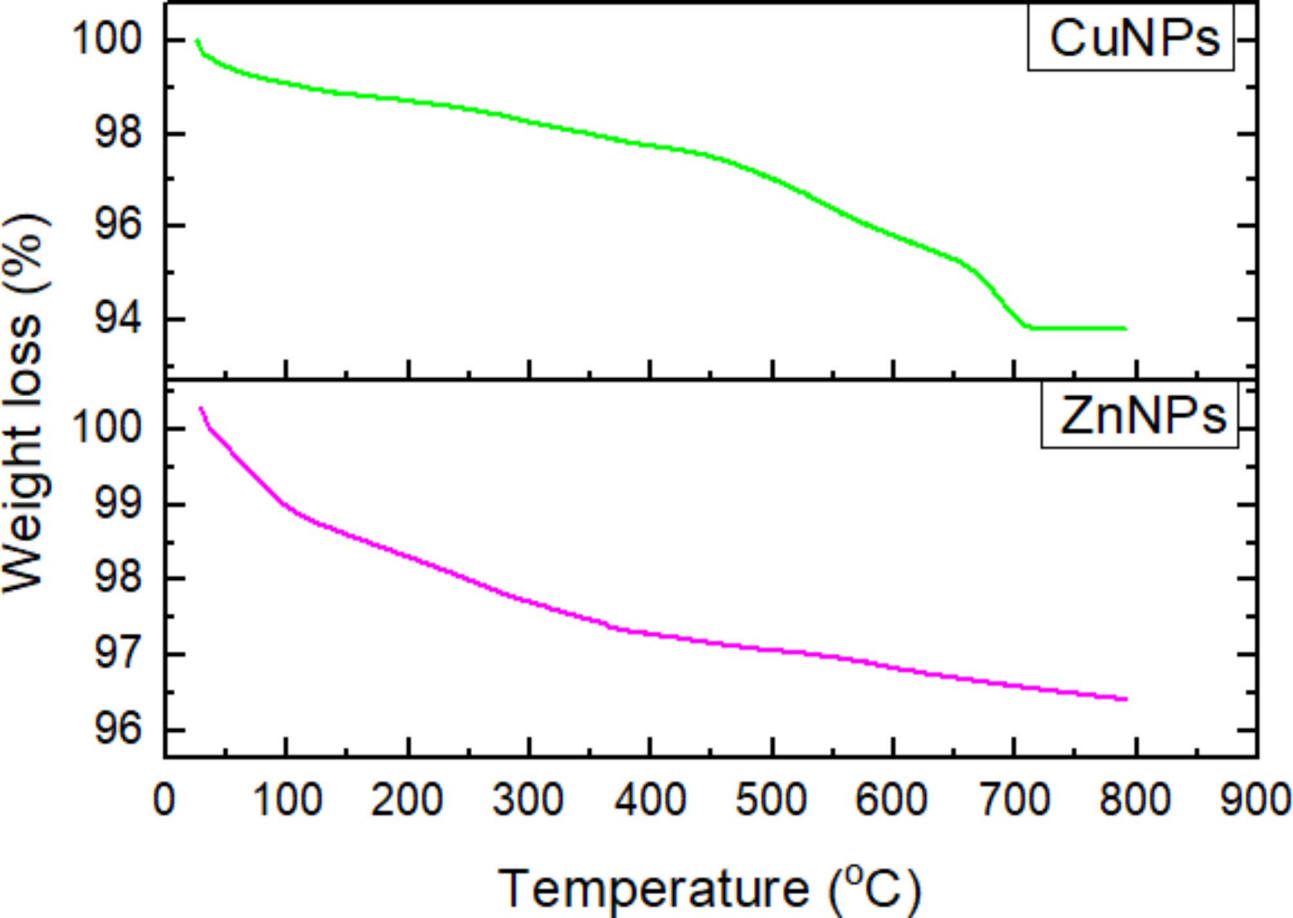
Thermogravimetric analysis of ZnO and CuO NPs.

In Fig. 3, the thermogravimetric analysis (TGA) revealed a two-stage weight loss pattern for both ZnO and CuO NPs, with losses of 4% and 6% observed at distinct temperature ranges. This multi-step decomposition process provides valuable insights into the composition and stability of the synthesized nanoparticles. The initial weight loss, occurring between 50 °C and 400 °C, can be attributed to two primary factors: The evaporation of physically adsorbed water molecules on the surface of the nanoparticles. This surface-bound water is loosely attached and readily desorbs at relatively low temperatures and the volatilization of highly volatile chemical components present in the sample. These may include residual solvents or unreacted precursors from the synthesis process. The subsequent weight loss, observed in the temperature range of 400 °C to 700 °C, is more significant and can be linked to the thermal decomposition of bioorganic components. These organic materials are likely derived from the plant extract used in the green synthesis of Zn and CuO NPs^37^. The plant extract serves as both a reducing agent and a capping agent during the nanoparticle formation, and its residual presence contributes to the observed weight loss at higher temperatures. The enhanced thermal stability can be attributed to strong interactions between the hydroxyl functional groups present in the plant extract and the metal oxide nanoparticles^38^.

#### Transmission electronic microscope

**Fig. 4:**
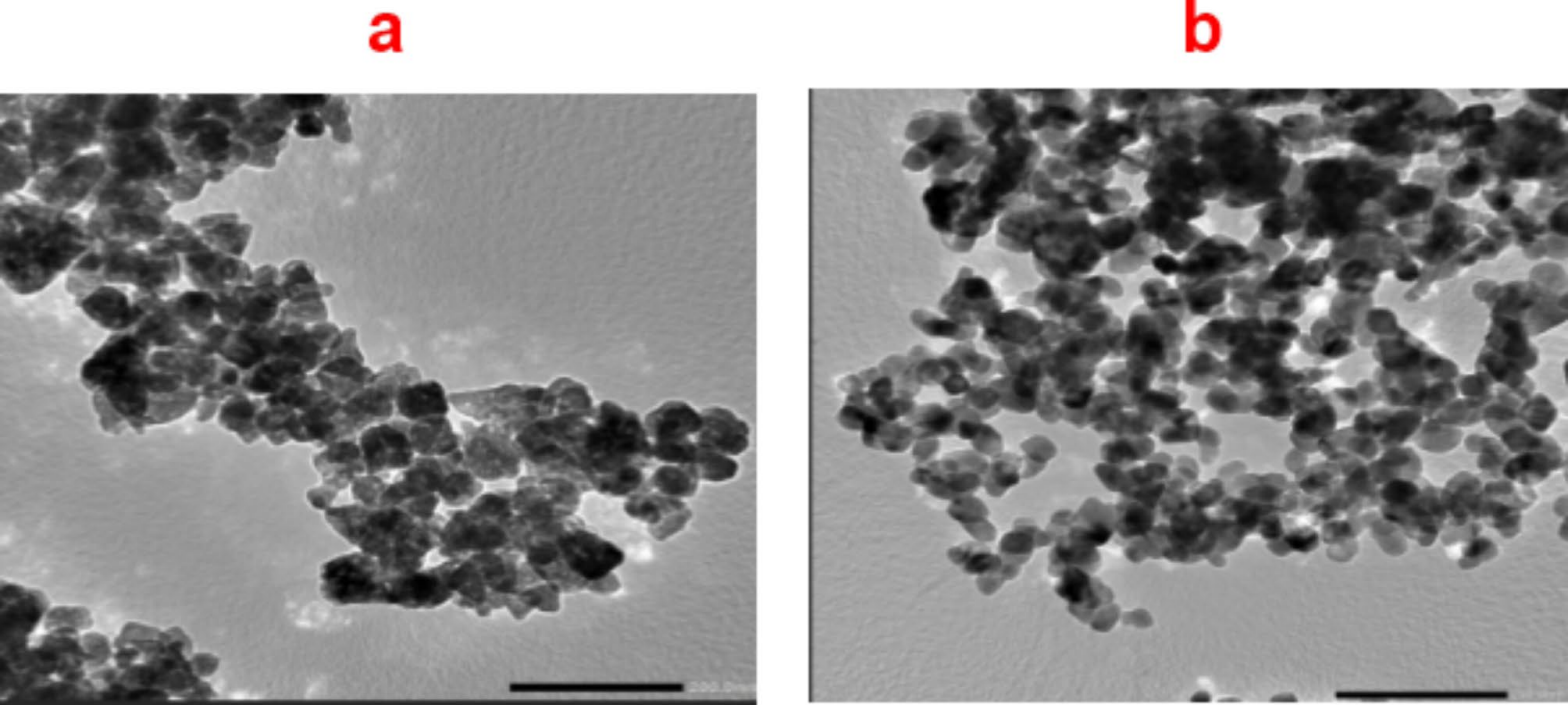
TEM images of (**a**) ZnO and (**b**) CuO nanoparticles.

Figure 4 illustrates a variety of morphologies observed in the synthesized nanoparticles, prominently featuring spherical and hexagonal shapes, as well as aggregates. Notably, the spherical particles are predominant in both cases examined. The size distribution of the ZnO nanoparticles ranges from 20 to 40 nm, while the CuO nanoparticles exhibit a smaller size range of 10 to 25 nm^37,39^. This size variation is significant because it can influence the physical and chemical properties of the nanoparticles, including their reactivity, surface area, and potential applications in fields such as catalysis and drug delivery. These findings presented here are consistent with previous research that has investigated copper oxide nanoparticles synthesized using plant extracts, specifically Magnolia champaca. Studies^40,41^ have demonstrated that plant-mediated synthesis not only affects the morphology but also enhances the stability and biocompatibility of metal oxide nanoparticles. The use of natural extracts for nanoparticle synthesis is gaining traction due to its eco-friendly approach compared to traditional chemical methods.

#### Hydrodynamic size, and zeta potentials

**Fig. 5:**
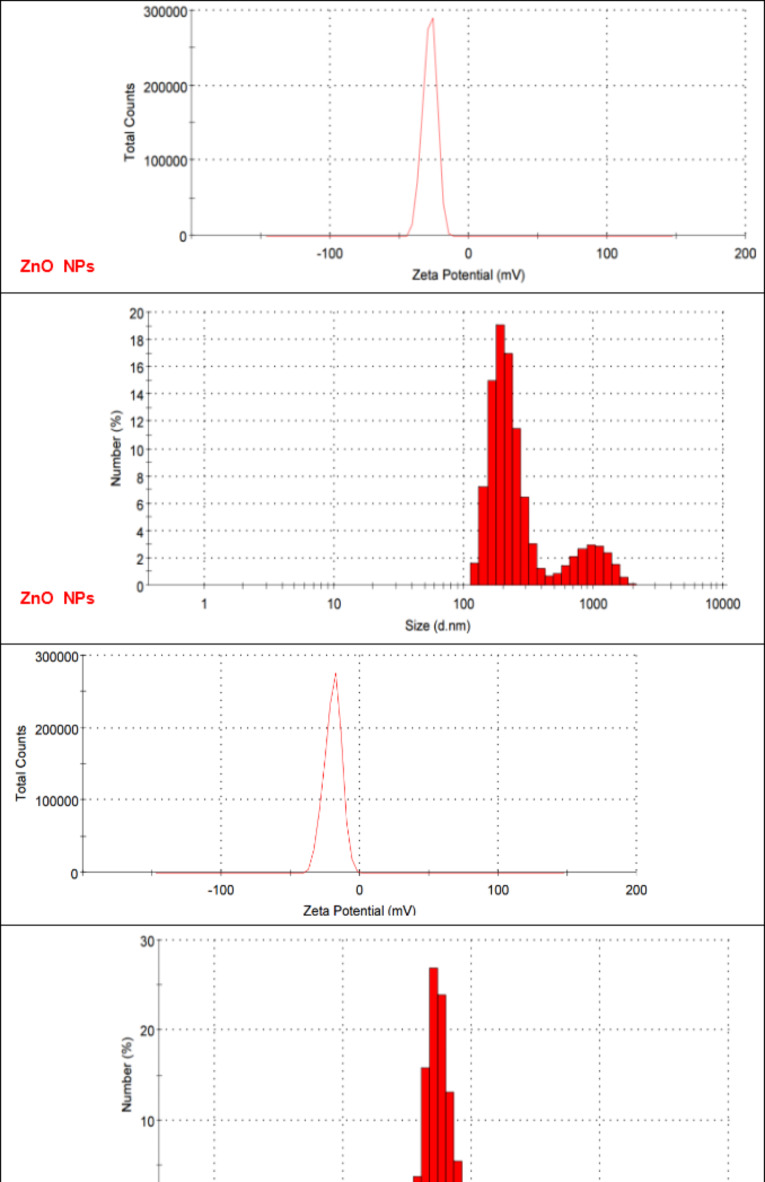
Zeta potential and hydrodynamic size distribution of ZnO and CuO NPs.

As shown in Fig. 5, the average hydrodynamic size of ZnO NPs and CuO NPs was 834 nm and 318 nm, respectively. The variation in size of NPs is attributed to the presence of polyphenolic compounds which have strong attractive forces between and holds the particles together which results in the formation of particles with variable size. Agglomerates formation is particularly due to the presence of intermolecular hydrogen bonding between hydroxyl groups of different phenolic compounds^42^.

Zeta Potential analysis is used to quantify the overall surface charge of the NPs and reflects the colloidal stability of nanoparticles. According to previous literatures, zeta potential value < − 30 mv or > + 30 mv forms stable colloidal suspension^43^. The zeta potential values of ZnO and CuO NPs were − 28.3 and − 19.7 respectively. The magnitude of zeta potential value reaches the maximum when formation of small-sized ZnO and CuO NPs is complete. This can also be correlated to the fact that the crystallite size decreases at phase of optimal growth; consequently, leading to an increase in the overall surface charge. The formation of any unstable aggregates in the reaction leads to a drop in the Zeta Potential. Therefore, good stability of the synthesized NPs is indicative of the absence of well-dispersed NPs without aggregates formation^44,45^.

### Pigment and paint evaluations

The characteristics of the pigments, as measured in accordance with ASTM standards, are summarized in Table 3.

**Table 3:** Physical properties of prepared ZnO and CuO NPs as pigments.

Property	ZnO NPs	CuO NPs
Bleeding (various solvents)	None	None
Fineness (Hegman units)	8 H	5 H
Moisture (%)	0.2	0.4
Oil Absorption (g/100 g)	115	75
pH	11	8
Loss on Ignition (%)	0	0

*Note* L.O.I = Loss on Ignition, H = Hegman (fineness unit), The various solvents including Ethylene Glycol, Toluene, Butyl Glycol, Normal Butanol, Methyl Ethyl Ketone. Dichloromethane, Chloroform, and Carbon tetrachloride.

So, based on Table 3, the following results are observed:

#### Hydrogen ion concentration (pH)

The pH values of the prepared pigments are 11 for ZnO, indicating a strongly alkaline nature, and 8 for CuO, indicating a slightly alkaline nature. These results are consistent with the literature.

#### Oil absorption

Oil absorption is an indicator of the amount of binder required to achieve full wetting of the pigment and form a uniform paint film. The ZnO NPs exhibited a high oil absorption value of 115, while CuO NPs showed a lower value of 75, suggesting that ZnO NPs will require more binder when used in paints.

#### Moisture content

The low moisture content in both ZnO and CuO NPs suggests that moisture has minimal impact on the pigments’ weight, both before and after their use in paint formulations.

#### Fineness of grind

The “fineness of grind” refers to the dispersion level of the pigment in a vehicle system, typically seen in liquid coatings. The results indicate that ZnO NPs (8 H) have a finer grind than CuO NPs (5 H), suggesting better dispersion of ZnO NPs in pigment-vehicle systems.

#### Bleeding of pigments

The bleeding test evaluates the color stability of a pigment when it comes into direct contact with various solvents. This is a quick and straightforward method for assessing the pigment’s resistance to bleeding. The data in Table 3 show that the prepared ZnO and CuO nanoparticles exhibit no bleeding (non-precipitable color), indicating a high level of stability in the coatings. The results further suggest that the prepared pigments have a high degree of stability, maintaining their color and form under exposure to light and heat, which indicates their excellent resistance to environmental factors.

#### Loss on ignition

This test measures the loss of pigment weight when subjected to high temperatures. The results show that both ZnO and CuO NPs maintain their weight and color under high temperatures, suggesting excellent thermal stability.

### Characterization of the prepared paint formulation based on the prepared ZnO and CuO NPs

#### SEM of the paint formula based on ZnO and CuO NPs

**Fig. 6:**
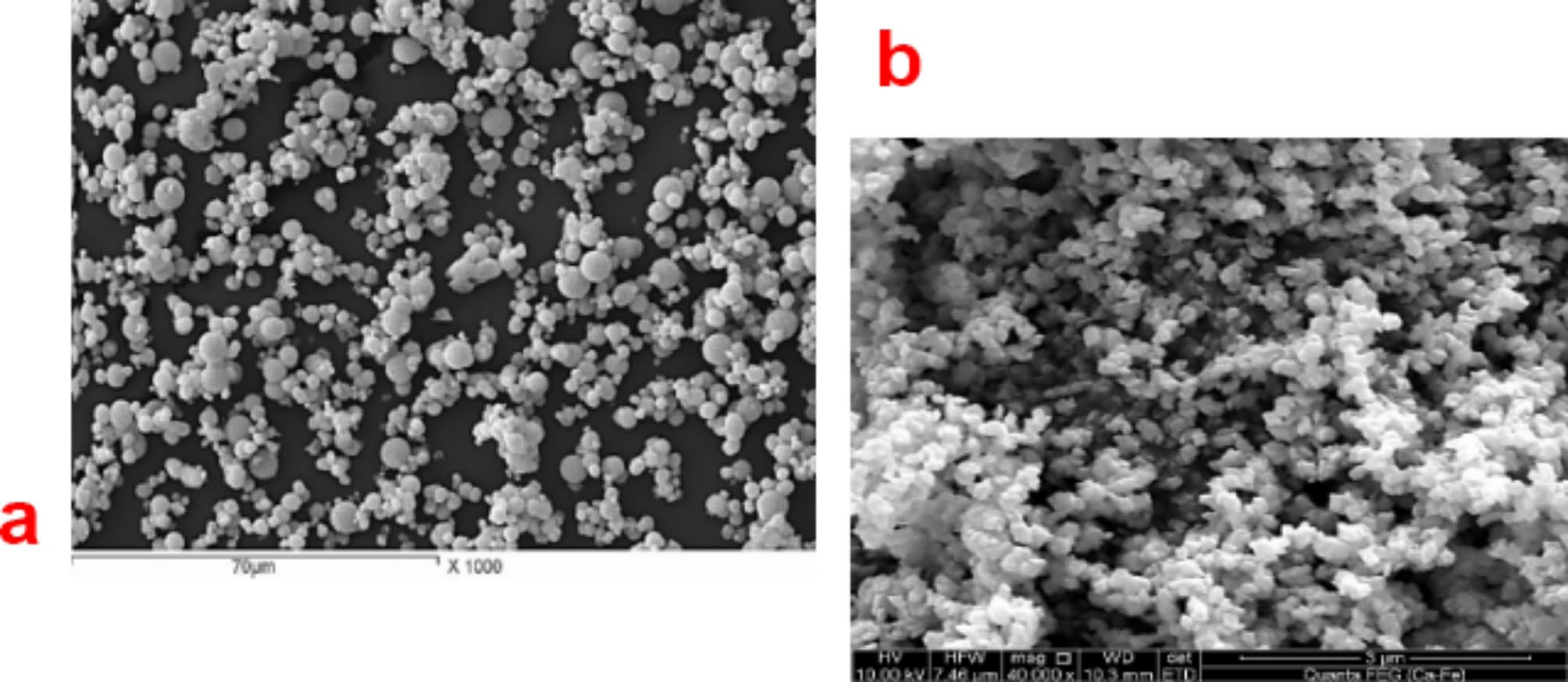
SEM images of the paint formula based on (**a**) ZnO and (**b**) CuO NPs.

In Fig. 6, there are no morphological irregularities. This indicates that the dispersion of ZnO NPs and CuO NPs in pigment-vehicle are good dispersion bat may be ZnO NPs was more dispersed than CuO NPs and this agreement with the obtained result of oil absorption and fineness of grind for both prepared metal oxides NPs.

### Mechanical properties of dry painted films

The physical and mechanical properties of the dry paint film from the prepared sample were evaluated and summarized in Table 4. Notably, films based on the prepared oxide nanoparticles exhibited excellent performance. These coatings, applied at a thickness of 80 ± 5 μm to mild steel strips using a spray method, showed no observable changes during testing.

Interestingly, the composition of the coating was unaffected by using different prepared pigment NPs. Films based on silicon resin demonstrated higher impact resistance and ductility, attributed to silicon’s elastic properties that enhance atomic movement within the film. Additionally, the integration of ZnO and CuO NPs into silicon cavities contributed to good film hardness, preventing defects that could lead to damage.

The chemical resistance of the dry-painted film, based on the prepared ZnO and CuO NPs, was evaluated against various solutions (Na_2_CO_3_, H_2_O, and H_2_SO_4_ mineral turpentine) as detailed in Table 4. Coated glass plates were submerged in these solutions for 48 h after curing. Remarkably, the films exhibited good resistance to acid. Glass plates submerged in distilled water, alkali, and solvent showed no change, likely due to their elevated hydrophobicity and the presence of prepared metal oxide NPs^12,18–20^.

**Table 4:** Physical, mechanical properties and chemical resistance of dry painted films based on ZnO, CuO NPs.

Composition	Impact (Kg)	Adhesion	Pending	Dry film thickness	Acid	Alkali	Solvent and Water
Formulation based on ZnO NPs	D = 1.5	5B	Pass	80 ± 5 μm	Good	Pass	Pass
Formulation based on CuO NPs	D = 1.4	5B	Pass	80 ± 5 μm	Fair	Pass	Pass

### Heat resistance test

**Fig. 7:**
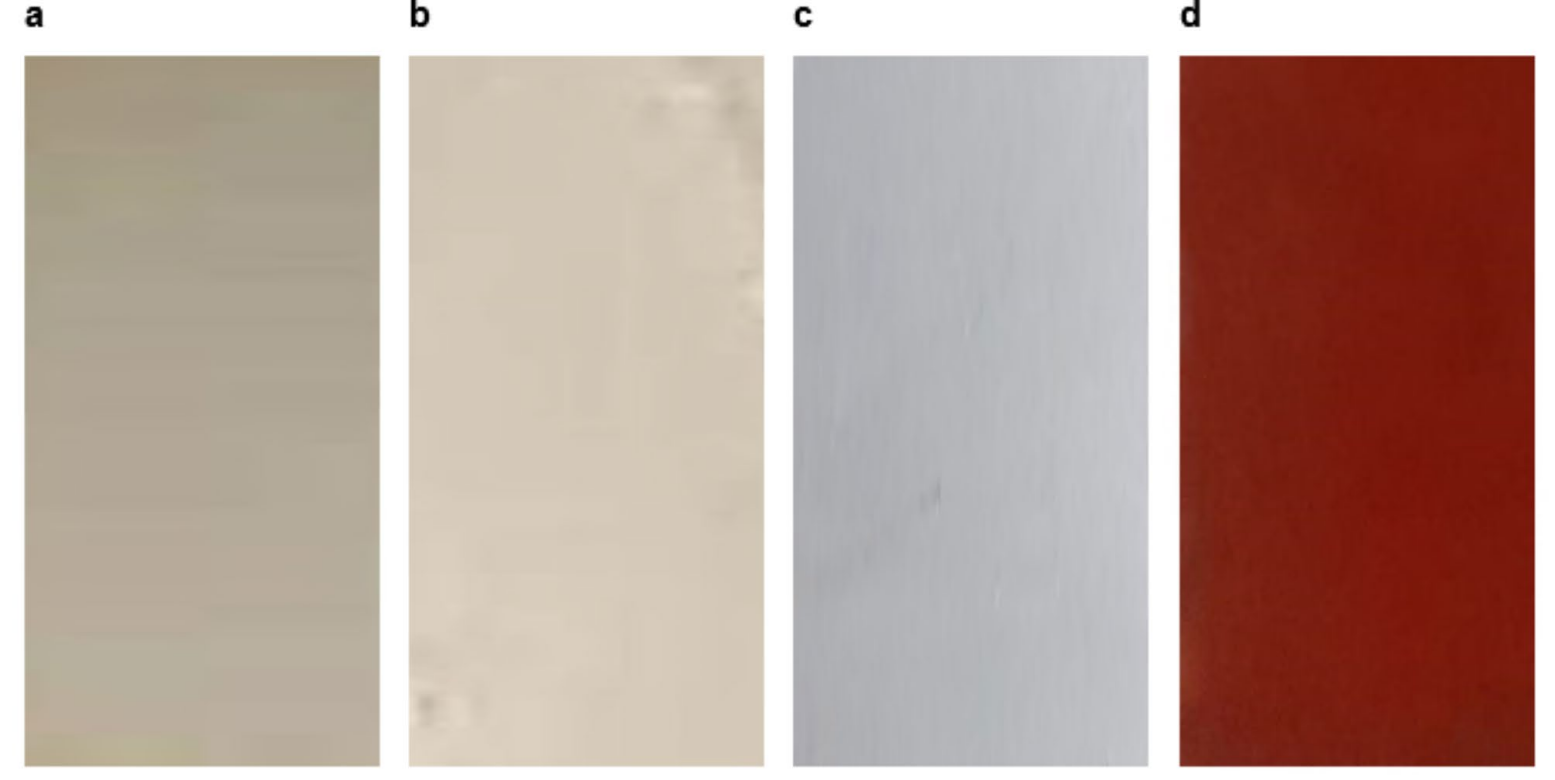
The photographic of the steel painted films before exposer to heat resistance test. (**a**) Painted film of based on F1 before exposure to heat, (**b**) based on F2, (**c**) based on F3, (**d**) based on F4.

**Fig. 8:**
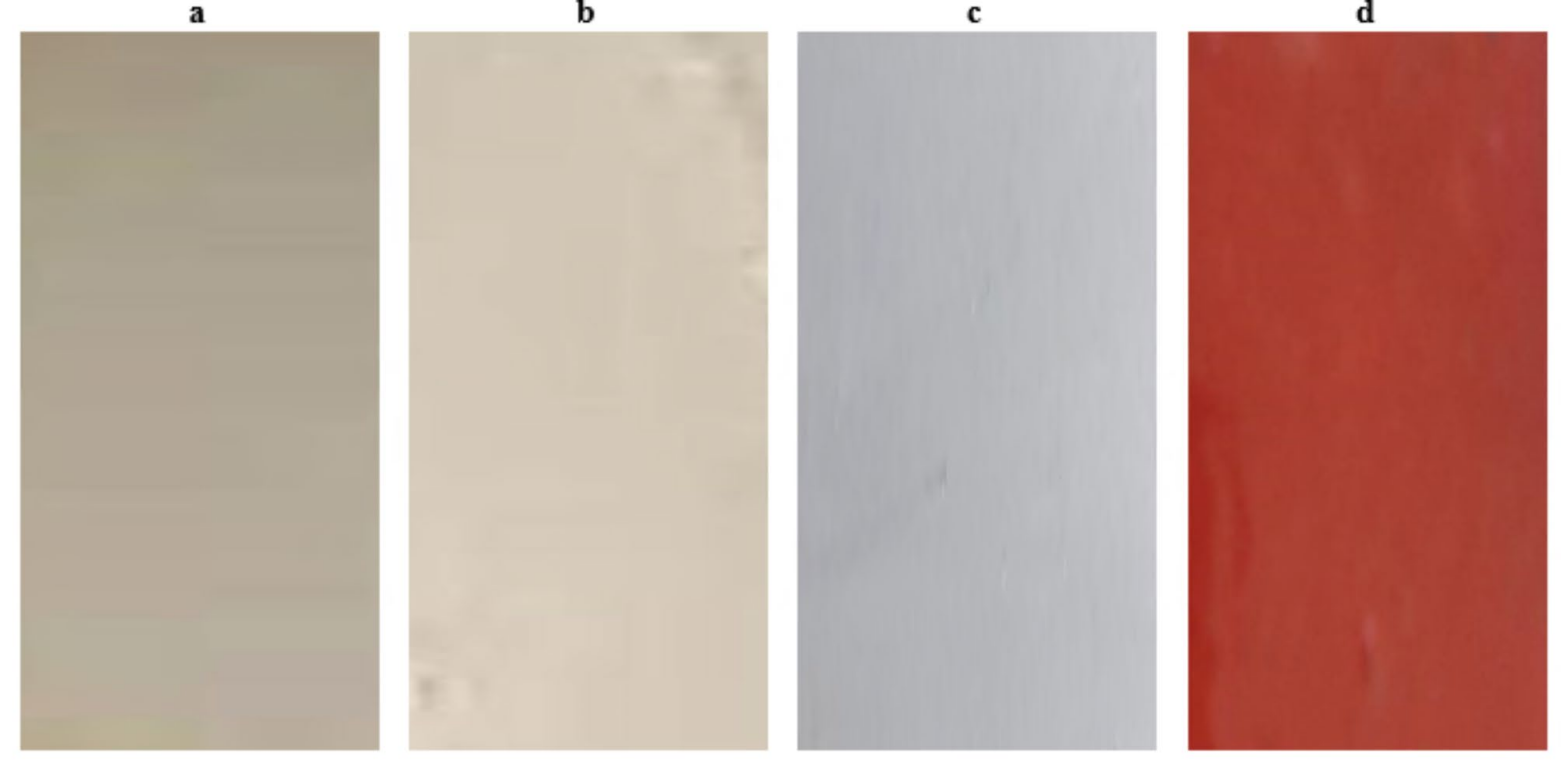
The photographic of the testes heat resistance of all the steel painted films. (**a**) Painted film of based on F1 After (6 h) exposure to heat at 500 °C (**b**) based on F2, (**c**) based on F3 and (**d**) based on F4.

These test techniques assess the heat-resistant qualities of coatings specifically formulated to safeguard steel surfaces that are subjected to high temperatures during their operational lifespan. We assessed the resistance to high temperature by visually comparing the surface with photographic reference standards that were also used. One important consideration is the degree of cracking and the change of color. So, based on the examination of these tested painted samples, we have observed that there is no damage, no cracking, and more uniform films and the same color before and after exposure to different elevated temperatures until 500 °C for both painted samples based on zinc phosphate (F2) and ZnO (F3), or CuO (F4) NPs as represented in Figs. 7 and 8, comparing with the blank or control sample (F1), this attributed to the degree of thermal stability can be ordered as: painted based on CuO > painted panels based on ZnO NPs. > painted panels based onzinc phosphate pigment. And this agreement with the loss on ignition test which has been done for the prepared metal oxides NPs before their using as a pigment in the paint formulation, which confirmed that there is no change in the weight and color for the prepared ZnO and CuO NPs, comparing with the control sample based on F1.

Also, the good results of painted steel panels based on F4, F3 and F2 may be attributed to the superior heat resistance of silicon compared to organic resins is its high thermal stability. This is attributed to the higher bond strength of Si-O, which is measured at 445 kJ mol^−1^. Consequently, more energy is required to disrupt this bond. The good results of heat resistance of all coated films containing (ZnO, CuO NPs) also, may be because of the good integration of ZnO and CuO into the cavities of silicon can hinder any defect that leads to cracking and damage. in addition to the high hiding power and best performance of the prepared ZnO and CuO NPs compared with sample blank and paint based on F2^18,20^.

### Corrosion resistance

**Fig. 9:**
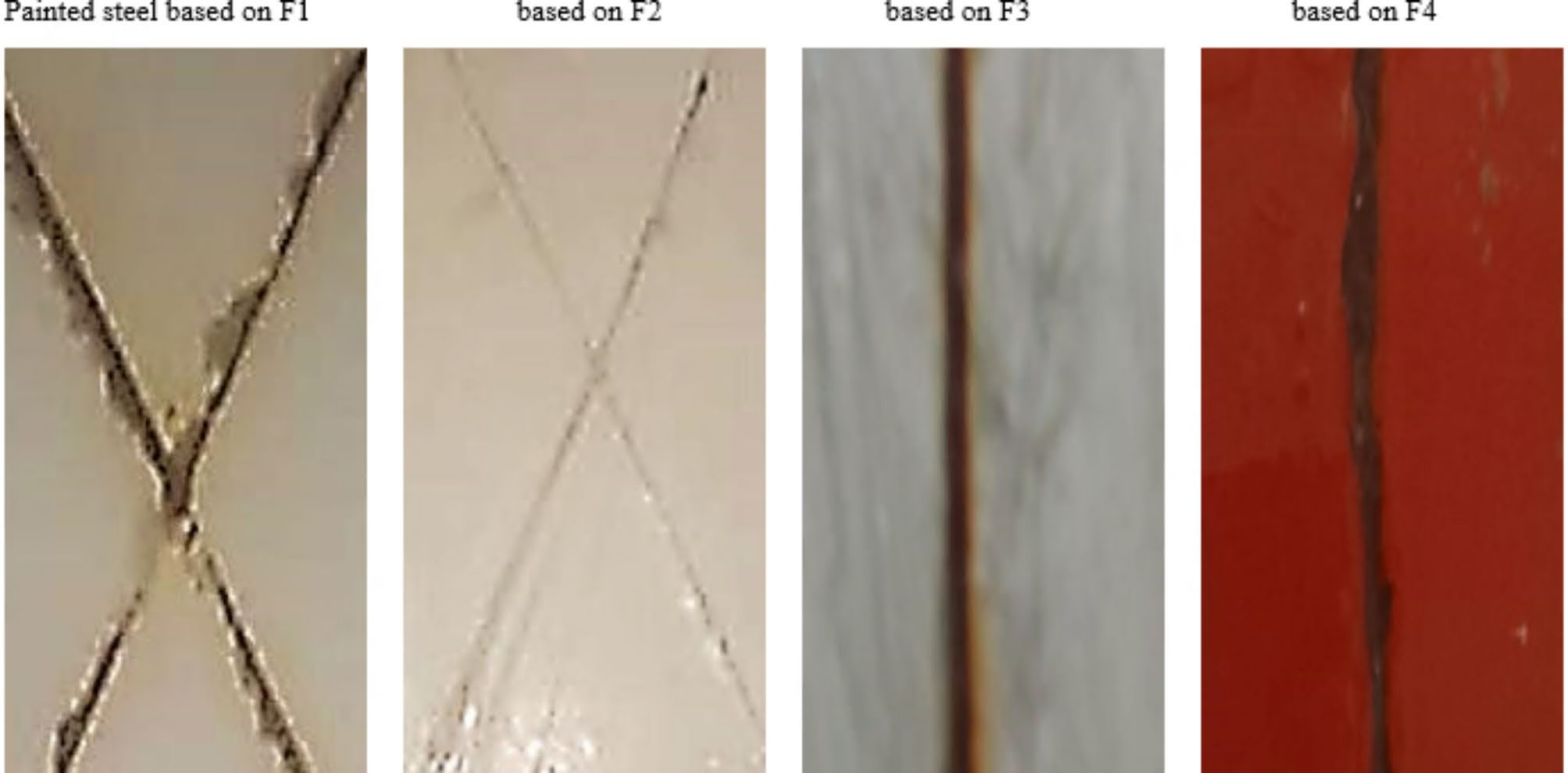
Photograph of the painted film after salt spray test (5% of NaCl). Where, F1: sample blank (without inhibitors). F2: standard control containing corrosion inhibitor zinc phosphate). F3: paint formula based on ZnO NP. F4: Paint formula based on CuO NPs.

Table 5 presents data and statistical information related to the salt spray test. After the films did not adhere properly, the salt spray test was stopped after 500 h exposer Fig. 9, visually presents the salt spray test results, featuring images of dried paints formulated with zinc phosphate as anticorrosive pigment (F2), ZnO (F3) or CuO nanoparticles (F4), as pigments or without pigment but based on the talc as filler (F!). Notably, the painted steel panels based on the prepared CuO and ZnO NPs were nearly similar with the results of painted steel panels based on zinc phosphate as a standard control formula, and the both formulations were noticed more efficiency than painted steel panels based on talc (F1), this results were agreement with the literature survey which confirmed that paint formulation based on zinc phosphate or zinc oxide are good choose for steel protection against corrosion because of the presence of zinc metal which enhance the corrosion resistance and protect and prevent the ions from the attack steel substrate.

**Table 5:** Evaluation the corrosion resistance of the painted films.

Formulation no	Blistering		Scribe Failure (mm)	Rust grade
	Size	Frequency		
Blank (F1)	4	P	7	3
Formulation based on zinc phosphate (F2)	9	F	2	9
Formulation based on ZnO NPs (F3)	9	F	2	8
Formulation based on CuO NPs (F4)	7	F	3	6

The high hiding power of the prepared ZnO and CuO NPs can develop the opacity in paint systems due to the nanoparticle, and also the best performance of prepared pigments are increase the efficiency the corrosion resistance comparing with the blank formula (F1) and F2.

Also, strong adhesion of the painted steel substrate based on the F2, F3 and F4 prevents moisture vapor from penetrating the coating and condensing in low-adhesion areas, which could lead to blistering. This consideration is crucial when selecting coating systems. The distribution of ZnO and CuO nanoparticles blended with silicon resin significantly impacts corrosion protection. Gradual incorporation of ZnO and CuO NPs reduces blister density and spot rusting. Overall, good corrosion protection and performance are linked to well-dispersed metal oxide NPs within the silicon polymer matrix. This improved adhesion acts as a barrier, isolating mild steel from corrosion due to its impermeability to water and corrosive ions^46^.

## Conclusion

This study successfully synthesized zinc and copper oxide nanoparticles using eco-friendly plant extract methods. Comprehensive analyses confirmed the formation of nanoparticles, demonstrating their potential as effective pigments. The incorporation of these nanoparticles into paint formulations, alongside silicon resins, resulted in films with excellent physico-mechanical properties, chemical and heat resistance, and corrosion resistance. Notably, ZnO nanoparticle-containing films exhibited superior performance in a 500 h salt spray test compared to those with CuO nanoparticles. These promising results highlight the potential of these synthesized mixed oxide nanoparticles for use in heat-resistant pigment applications.

## Data Availability

The datasets used and analyzed during the current study are available from the corresponding author upon reasonable request.
